# The in vivo impact of MsLAC1, a *Miscanthus* laccase isoform, on lignification and lignin composition contrasts with its in vitro substrate preference

**DOI:** 10.1186/s12870-019-2174-3

**Published:** 2019-12-12

**Authors:** Feng He, Katja Machemer-Noonan, Philippe Golfier, Faride Unda, Johanna Dechert, Wan Zhang, Natalie Hoffmann, Lacey Samuels, Shawn D. Mansfield, Thomas Rausch, Sebastian Wolf

**Affiliations:** 1 Centre for Organismal Studies (COS) Heidelberg, Heidelberg University, Heidelberg, Germany; 2 Department of Wood Science, University of British Columbia, Vancouver, Canada; 3 Department of Botany, University of British Columbia, Vancouver, Canada

**Keywords:** *Miscanthus*, *Arabidopsis*, Laccase, Lignin, S/G ratio, Interfascicular fibers, Lignocellulosic biomass

## Abstract

**Background:**

Understanding lignin biosynthesis and composition is of central importance for sustainable bioenergy and biomaterials production. Species of the genus *Miscanthus* have emerged as promising bioenergy crop due to their rapid growth and modest nutrient requirements. However, lignin polymerization in *Miscanthus* is poorly understood. It was previously shown that plant laccases are phenol oxidases that have multiple functions in plant, one of which is the polymerization of monolignols. Herein, we link a newly discovered *Miscanthus* laccase, MsLAC1, to cell wall lignification. Characterization of recombinant MsLAC1 and *Arabidopsis* transgenic plants expressing MsLAC1 were carried out to understand the function of MsLAC1 both in vitro and in vivo.

**Results:**

Using a comprehensive suite of molecular, biochemical and histochemical analyses, we show that MsLAC1 localizes to cell walls and identify *Miscanthus* transcription factors capable of regulating *MsLAC1* expression. In addition, *MsLAC1* complements the *Arabidopsis lac4–2 lac17* mutant and recombinant MsLAC1 is able to oxidize monolignol in vitro. Transgenic *Arabidopsis* plants over-expressing *MsLAC1* show higher G-lignin content, although recombinant MsLAC1 seemed to prefer sinapyl alcohol as substrate.

**Conclusions:**

In summary, our results suggest that *MsLAC1* is regulated by secondary cell wall MYB transcription factors and is involved in lignification of xylem fibers. This report identifies *MsLAC1* as a promising breeding target in *Miscanthus* for biofuel and biomaterial applications.

## Background

*Miscanthus*, a perennial C4 grass, has been promoted as a promising energy crop due to its high biomass yield and low demand for fertilizer and pesticides [1]. Compared to other species, *Miscanthus* biomass contains significantly less moisture and produces less ash, making it suitable for biofuel generation and production of valuable chemicals via bio-conversion processes [2]. However, current *Miscanthus* biomass utilization for biofuel production is largely limited by cell wall recalcitrance towards biochemical conversion, to which lignin content and quality contributes significantly. In addition, lignin decreases digestibility when *Miscanthus* biomass is used as feed [3]. Consequently, lignin content and quality as well as the cellulose-to-lignin ratio have a substantial impact on the utilization and degradability of *Miscanthus* biomass [3, 4]. Therefore, a deeper understanding of factors controlling lignin biosynthesis and deposition in *Miscanthus* is required for improving the utility of this abundant source of lignocellulosic biomass.

Lignin is one of the major components of plant secondary cell walls and is mainly composed of the polymerized monolignols *p*-coumaryl alcohol, coniferyl alcohol, and sinapyl alcohol, resulting in H, G, and S lignin, respectively. Lignin biosynthesis includes three major steps: monolignol biosynthesis in the cytosol, transport of monolignols to the cell wall matrix, and polymerization into the heterogeneous, cross-linked lignin polymer [5]. While recent studies in *Arabidopsis* have identified and characterized a large number of enzymes and transcription factors responsible for these steps, and each can significantly affect lignin content and composition [6], knowledge about lignification in *Miscanthus* remains thus far limited. However, a recently completed transcriptome analysis, based on developing internodes of *Miscanthus lutarioriparius*, a Miscanthus transcriptome database [7], and *Miscanthus sinensis* draft genome (*Miscanthus sinensis* v7.1 DOE-JGI, http://phytozome.jgi.doe.gov/), revealed many similarities to other species concerning the secondary cell wall biosynthetic machinery [8]. Furthermore, the transcription factor MsSND1 was shown to be able to regulate secondary cell wall formation, including lignification, similar to its orthologue AtSND1 in *Arabidopsis* [9]. These results and previous studies in other monocots indicate a partially conserved secondary cell wall biosynthetic pathway between monocot and dicot plants [10–13]; allowing the study of potential lignification-related genes of *Miscanthus* by exploiting existing knowledge from *Arabidopsis* and other plant species.

Following biosynthesis, monolignols are oxidized by peroxidases and/or laccases to monolignol radicals, which spontaneously polymerize in the cell wall.. There is strong experimental support for peroxide-dependent peroxidases [14] and oxygen-dependent laccases in the polymerization process [15–18]. In *Arabidopsis*, laccase isoforms involved in lignification appear to have redundant functions [19]. Specifically, the *Arabidopsis lac4* or *lac17* single mutants result in only slightly reduced lignin content, whereas the *lac4–2 lac17* double mutant displays up to 40% less lignin in the stem [15]. Furthermore, the *lac4 lac11 lac17* triple mutant is characterized by vascular bundles almost entirely devoid of lignin [19]. Although attempts have been made to identify the physiological role of different laccase isoforms [18, 20], it remains difficult to assign specific functions to individual laccases due to redundancy and broad substrate specificities [16].

In this study, a *Miscanthus* laccase isoform closely related to *AtLAC17* was cloned and named *MsLAC1*. *MsLAC1* transcripts were expressed primarily in elongating internodes, sharing an expression pattern with other secondary cell wall-related genes. Further experiments revealed that the promoter of the *MsLAC1* gene is targeted by MsSCM4, a putative orthologue of the *Arabidopsis* secondary wall synthesis regulators AtMYB58/63 [9]. MsLAC1 protein is secreted into the cell wall and the recombinant MsLAC1 protein is able to catalyze oxidation of monolignols. *MsLAC1* also functionally complements the *Arabidopsis lac4–2 lac17* double mutant, and upon ectopic expression impacts lignification, both in quantity and quality, with an increased S/G ratio.

## Results

### Identification of laccase sequences in a *Miscanthus sinensis* transcriptome

**Fig. 1:**
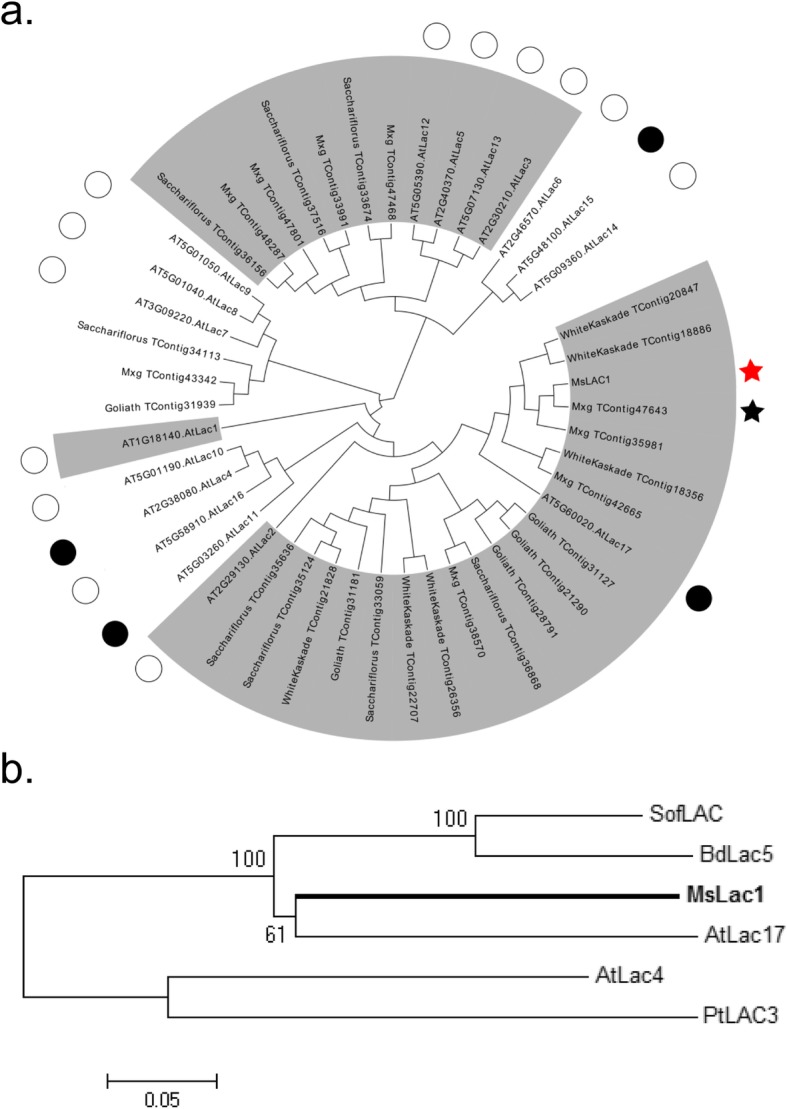
Phylogenetic analysis of laccase sequences from different plant species. Alignments were conducted with Mega 5 using the Neighbor-joining method. **a** All available *Miscanthus* laccase sequences from different species (e.g. *goliath*, *saccharifolius*) and *Arabidopsis* laccases (marked by circle) were included. Black circles indicate *Arabidopsis* laccases involved in lignin biosynthesis. Different laccase subgroups were categorized based on the results of (Turlapati et al., 2011) and marked with grey background. *Mxg_TContig47643* (black star), which clustered in subgroup 1 together with *AtLAC17*, was used for primer design to amplify the open reading frame of *MsLAC1* (red star). Note that *MsLAC1* is closely related to, but not identical with the open reading frame of *Mxg_TContig47643* (95% sequence identity at protein level). **b** Sequences of *Miscanthus* (*MsLAC1*), *Brachypodium* (*BdLAC5*), *Saccharum* (*SofLAC*), *Populus* (*PtLAC3*) and *Arabidopsis* (*AtLAC4* and *AtLAC17*) laccases were included

Using published laccase nucleotide sequences from *Arabidopsis*, *Sorghum*, and *Brachypodium*, we performed tBLASTn analysis of a *Miscanthus* transcriptome [7]. In total, 95 laccase-like contigs were identified, 28 of them containing complete sequences for putative laccase open reading frames (ORFs). Previous studies in *Arabidopsis* suggested a division of the laccase family into six subgroups [21], but fewer subgroups have been described for monocot species. For example, in *Brachypodium*, phylogenetic analysis revealed four subgroups [22], while sugarcane contains five subgroups [16]. Compared to dicot species, laccase families in monocots appear to be larger [16, 22]. A phylogenetic analysis was performed to classify laccase contigs found in the *Miscanthus* EST database [7] against *Arabidopsis* and *Brachypodium* laccases, respectively (Fig. 1 and Additional file 1: Figure S1).

This analysis identified *Mxg_TContig47643* (Fig. 1) as an ORF related to *AtLAC17*, which is known to be involved in lignification [15]. We cloned a laccase coding sequence, which we named *MsLAC1*, using primers based on *Mxg_TContig47643* from a of *Miscanthus sinensis* cDNA preparation. At the protein level, the sequence identities of MsLAC1 were 96 and 68%, compared to Mxg_TContig47643 and AtLAC17*,* respectively, indicating that *MsLAC1* was derived from a gene not identical but closely related to *Mxg_TContig47643*. To inspect the relationship between *MsLAC1* and other laccases that were previously reported to be involved in lignification, a multiple sequence alignment was carried out with characterized laccase proteins (Fig. 1b, Additional file 1: Figure S2), including those from *Brachypodium distachyon* (BdLAC5, [23]), sugarcane (SofLAC, [16]), poplar (PtLAC3, [17]), and *Arabidopsis thaliana* (AtLAC4 and AtLAC17, [15]). AtLAC17, which affects the deposition of guaiacyl (G) lignin in the interfascicular fibers [15], showed the highest sequence similarity to MsLAC1 (77%). MsLAC1 contains four conserved copper-binding sites and a predicted N-terminal signal peptide for secretion (Additional file 1: Figure S2). The predicted molecular weight and isoelectric point of non-glycosylated MsLAC1 are 63.0 kDa and 5.95, respectively. However, since the MsLAC1 protein sequence contains more than 15 putative N-glycosylation sites, the mature protein was expected to be considerably larger [24].

### *MsLAC1* is preferentially expressed in lignifying tissues, including young internodes and leaf sheaths and is co-expressed with other lignification genes

**Fig. 2:**
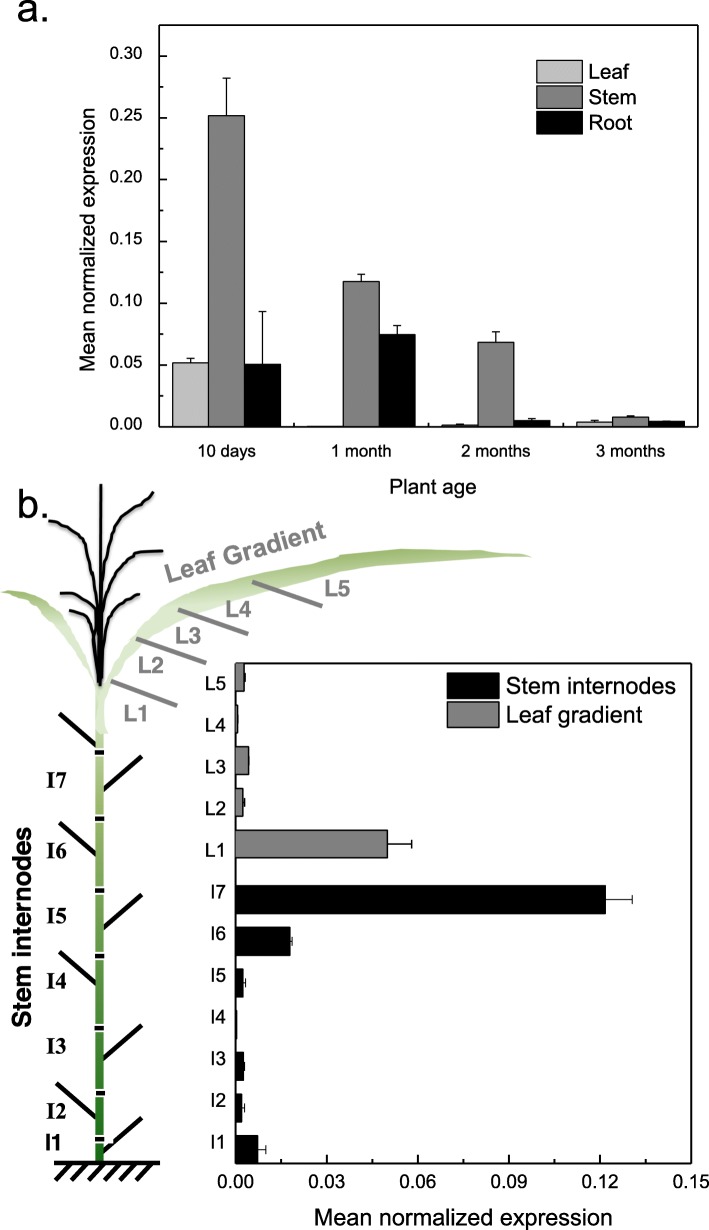
Expression pattern of *MsLAC1* as quantified by qPCR. Expression pattern in **a** different tissues of different plant age and **b** stem internodes and leaf gradient of mature, 6-month-old *Miscanthus* plants. The cartoon in **b** displays the sampling mode. Transcript levels were determined by qPCR and normalized with respect to PP2A expression

To explore whether MsLAC1 is involved in the lignification process, the relative expression levels of *MsLAC1* were first determined by qPCR in different organs (leaf, stem, and root) in plants of increasing age (10 days, 1 month, 2 months and 3 months; Fig. 2a). While *MsLAC1* transcripts were detected in all organs, the highest expression was detected in the stem, which is typically highly lignified. In general, the expression of *MsLAC1* decreased with increasing plant age. Since the process of lignification starts early during tissue maturation, *MsLAC1* transcripts were subsequently monitored along developmental gradients of stem and leaf, respectively. Specifically, seven shoot internodes as well as five leaf sections from 6-month-old, mature plants were analyzed for *MsLAC1* expression (Fig. 2b). In agreement with [22], the highest expression was detected in the youngest tissues, i.e., the leaf sheath (including the growing zone) and the youngest shoot internode, while gene expression was significantly lower in mature tissues.

**Fig. 3:**
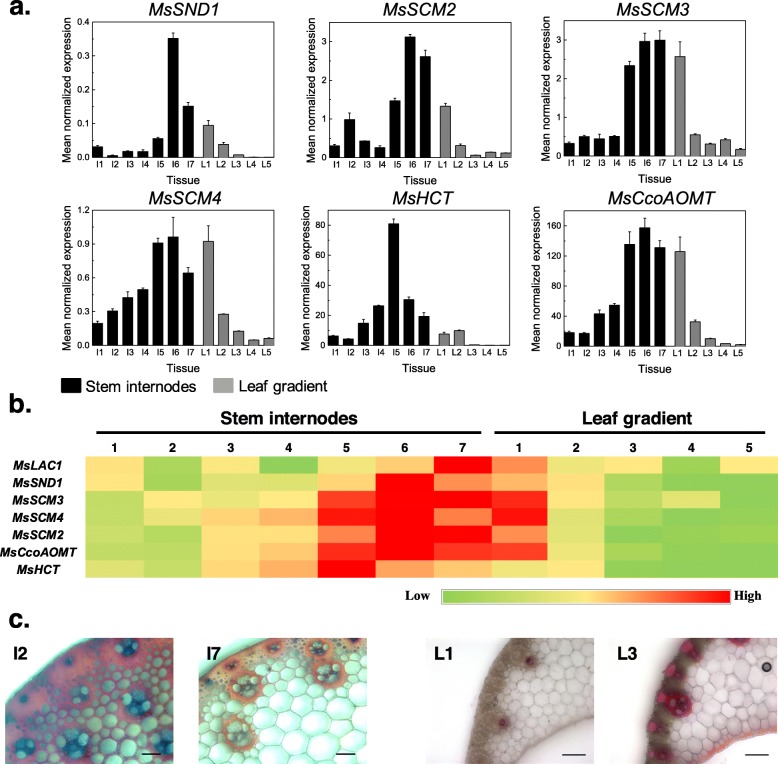
Expression pattern of different genes along developmental gradients of 6-month-old *Miscanthus* plants. **a** Transcript levels were determined by qPCR and normalized with respect to PP2A expression; **b** visualization via heat map for comparison of the expression patterns. **c** Cross sections of 2nd and 7th stem internodes and 1st and 3rd leaf gradient samples were stained with HCl-phloroglucinol. Scale bars: 100 μm. *MsSND1*, *M. sinensis SECONDARY WALL-ASSOCIATED NAC DOMAIN1*; *MsSCM2–4*, *M. sinensis SECONDARY CELL WALL MYBs 2–4*; *MsCcoAOMT*, *M. sinensis Caffeoyl-CoA O-methyltransferase*; MsHCT, *M. sinensis Hydroxycinnamoyltransferase*

To compare the expression of *MsLAC1* with genes predicted to be involved in lignification, co-expression analysis was performed. Expression of putative cell wall-related transcription factors *MsSND1*, *MsSCM2, 3,* and *4,* and two likely orthologues of genes involved in lignin biosynthesis, *MsCCoAOMT* and *MsHCT*, were compared with *MsLAC1* gene expression along the *Miscanthus* plant development gradients [9, 25]. Generally, all selected genes displayed higher expression levels in the stem as compared to the leaf (Fig. 3a). Along the leaf gradient, highest expression of the majority of genes was observed in the first section (i.e., including the sheath) of the leaf (or second for *MsHCT*), and expression levels decreased sharply in more mature leaf sections (Fig. 3a; b). For all analyzed genes, highest expression in the stem was detected in the three youngest internodes, strongly declining in older internodes, in agreement with lignin staining patterns (Fig. 3c). Together, these results are consistent with the hypothesis that *MsLAC1* is involved in the lignification process.

### The promoter of the *MsLac1* gene is activated by transcription factors regulating lignin biosynthesis

**Fig. 4:**
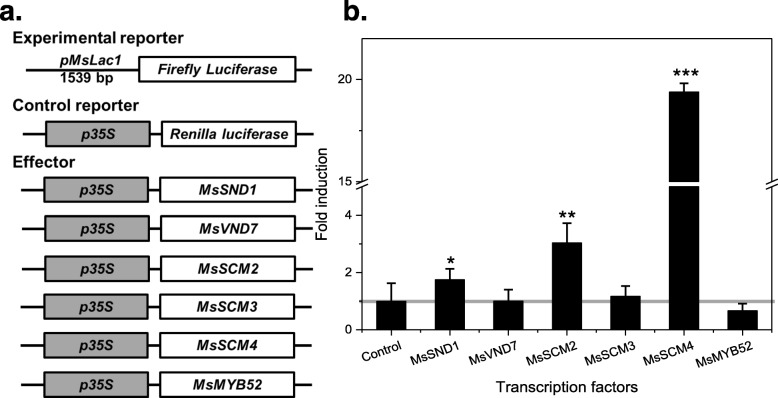
Activation of *MsLAC1* promoter by SND1 and SCM transcription factors from *M. sinensis*. **a** Constructs for dual luciferase assay; *Renilla* luciferase gene fused with 35S promoter was included for normalization. **b** Activation of *MsLAC1* promoter by different transcription factors. Gene constructs were delivered by particle bombardment to grapevine suspension cultured cells as previously described (Golfier et al., 2017); all experiments were repeated at least 3 times, and bars indicate mean ± SD of three technical replicates from a representative experiment. Student’s t-test was used to determine the significant difference of the induction: *, *P* < 0.05; **, *P* < 0.01; ***, *P* < 0.001

The newly identified sequence of *MsLAC1* was used to query the regulatory 5′-upstream region in a partially assembled genome sequence database of *Miscanthus sinensis*. A region comprising 1539 bp upstream of the *MsLAC1* start codon was isolated from genomic DNA. This putative promoter sequence was first analyzed for possible transcription factor (TF) binding sites, using the Genomatix MatInspector software, using a core similarity of > 0.85 [26]. Secondary cell wall NAC binding elements (SNBEs) and secondary wall MYB responsive elements (SMREs) are important cis-elements involved in the regulation of secondary wall biosynthesis by associated NAC and MYB factors [27]. Using the MatInspector software, a total of 16 putative regulatory sites were identified in the proposed regulatory region, including three SNBEs and two SMREs. The regulation of cell wall lignification is controlled via a complex network, including a number of well-characterized transcription factors, e.g. MYB factors [28]. To establish the corresponding network for *Miscanthus*, we had previously cloned *MsSND1*, *MsVND7*, *MsSCM2, 3, 4*, and *MsMYB52* as putative orthologues of the *Arabidopsis* secondary wall synthesis regulators *AtSND1*, *AtVND7*, *AtMYB85*, *AtMYB43*, *AtMYB58/63* and *AtMYB52*, respectively [9, 29]. We tested the ability of these *Miscanthus* TFs to regulate *MsLAC1* expression by quantifying the activity of a luciferase reporter driven by the *MsLAC1* promoter after co-bombardment into grapevine suspension-cultured cells (Fig. 4a). The results demonstrate that *pMsLAC1* was activated by MsSND1, MsSCM2 and MsSCM4 (Fig. 4b). The two MYB factors caused stronger activation than MsSND1, the highest induction (about 20-fold) being observed for MsSCM4. As reported earlier in *Arabidopsis*, AtMYB58 and AtMYB63 (homologs MsSCM4) act as direct transcriptional activators of lignin biosynthesis genes and are downstream targets of AtSND1 [30]. The current results suggest that *MsLAC1* may be a target of *Miscanthus* secondary cell wall-regulatory transcription factors.

### Transiently expressed MsLac1 localizes to the cell wall of tobacco epidermal leaf cells

**Fig. 5:**
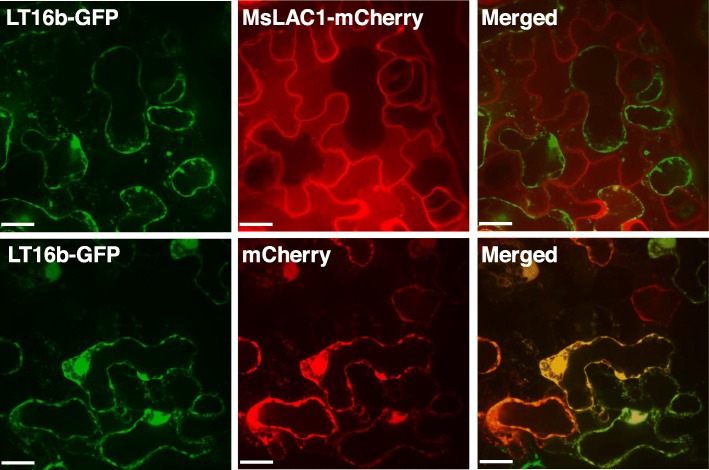
Localization of MsLAC1 in the cell wall compartment after transient expression in *Nicotiana benthamiana* leaf. Transient expression of MsLAC1-mCherry and LT16b-GFP (plasma membrane marker fused with GFP) in tobacco epidermal leaf cells after co-infiltration with A*grobacteria* harboring the respective constructs. Upper panel shows cell wall association of MsLAC1, whereas the Lti6-GFP-labelled plasma membrane retracts. Lower panel shows a cell co-expressing Lti6b-GFP and a cytosolic mCherry control retracting with the protoplast. All pictures were taken 2 h after onset of plasmolysis (i.e. incubation in 30% w/v sucrose solution). Scale bars: 16 μm

In *Arabidopsis*, AtLAC4, which contributes to stem lignification [15], was localized in the lignified secondary cell wall [31]. Sub-cellular localization of MsLAC1 was assessed by transient expression in tobacco leaves, using a fusion with a C-terminal pH-stable mCherry-tag [32]; Fig. 5). To distinguish between the plasma membrane and the cell wall, the plasma membrane-localized fusion protein LTI6b-GFP was co-transformed [33]. After plasmolysis, the GFP signal retracted with the cytosol, while MsLAC1-mCherry remained associated with the cell walls of the epidermal cells, thus indicating localization of MsLAC1 to the cell wall matrix (Fig. 5).

### MsLAC1 oxidizes monolignols in vitro

**Fig. 6:**
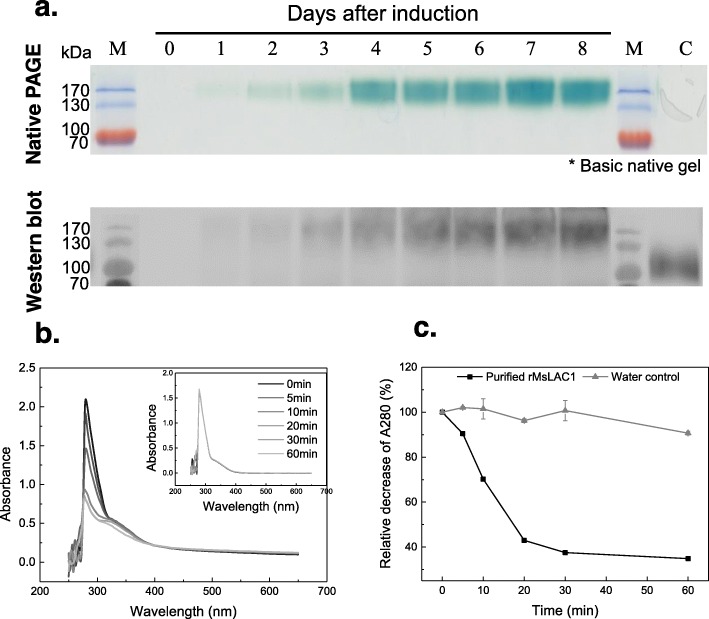
Expression of recombinant MsLAC1 protein and oxidation of sinapyl alcohol by purified recombinant protein. **a** The protein was expressed in *P. pastoris* and analyzed by Native PAGE and Western blot. After methanol induction samples of culture medium were collected at daily intervals, cleared by centrifugation, and 10 μl of supernatant were filtered and loaded. The zymogram was stained with 10 mM ABTS. Western blot was developed with anti-MYC antibody. M: PageRular Prestained Protein Ladder, 10–170 kDa. C: Crude extract from fermentation broth of *P. pastoris* transformed with pPICZαAfeh, 8 days after induction. Sinapyl alcohol (final concentration 1 mM) was incubated with 0.002 U recombinant MsLAC1 protein in 50 mM acetate buffer, pH 3.0. **b** Spectra of reaction mixtures (250 nm – 650 nm) were recorded at indicated time intervals (the insert indicates the control with substrate only). **c** Time course of absorption change at 280 nm. The initial absorbance at 280 nm was set as 100% individually for the calculation of absorbance decrease with purified protein and water control

To determine the enzymatic activity of MsLAC1, we generated recombinant protein in *Pichia pastoris*. Zymogram analysis revealed a time-dependent increase of a single activity band, reaching its maximum intensity 8 days after induction expression (Fig. 6a). Western blot analysis using anti-MYC antibody confirmed the increasing concentration of recombinant MsLAC1 protein with a signal at ~ 150 kDa (bottom panel, Fig. 6a). As indicated above, 15 putative N-glycosylation sites were predicted for the MsLAC1 protein sequence. Thus, the conspicuous difference between the calculated molecular mass for the MsLAC1 protein sequence (63 kDa) and the produced recombinant MsLAC1 (150 kDa) is likely due to N-glycosylation and, possibly, additional O-glycosylation or other post-translational modifications [24, 34, 35].

To understand the catalytic property of the recombinant MsLAC1, the oxidation of different monolignols were measured by determining the decrease of different substrates. While recombinant MsLAC1 enzyme was able to oxidize all supplemented monolignols, sinapyl alcohol was oxidized with the highest efficiency (Fig. 6). In contrast, cinnamyl alcohol was oxidized with the lowest efficiency (Additional file 1: Figure S3). Apparently, the efficiency of recombinant MsLAC1 enzyme was connected to the presence of methoxy substituents on the various substrates. Previous studies have also shown that a methoxy substituent in the ortho-position relative to the phenolic OH-group can enhance the polymerization rate by laccases [36]. These results suggest that the recombinant MsLAC1 is likely capable of oxidizing monolignols to radicals, which undergo cross-coupling spontaneously to form more complex polymers.

### Phenotypical complementation and recovery of both lignin content and lignin composition in the *Arabidopsis lac4–2 lac17* double mutant by MsLAC1

**Fig. 7:**
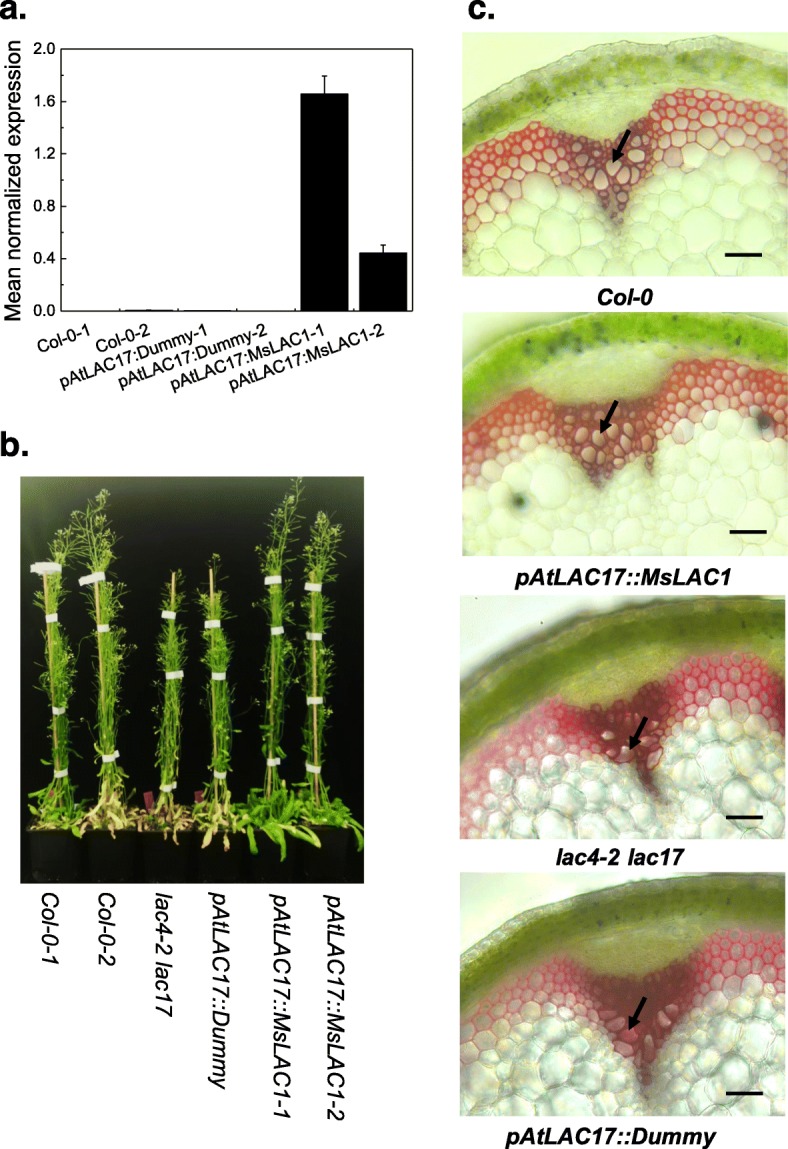
Complementation of *lac4–2 lac17* double mutant with *MsLAC1*, expressed under control of the *AtLAC17* promoter. **a** Confirmation of *MsLAC1* transcript expression in the complemented double mutant by qPCR (*AtPDF2* used as reference gene). **b** Phenotype of *Col-0* (wild type), double mutant *lac4–2 lac17*, and *pAtLAC17::MsLAC1*-complemented double mutant plants grown in growth chamber under constant light (135 μmol/m^2^s). **c** Stem cross-sections stained for lignification with HCl-phloroglucinol. Scale bars: 100 μm

To assess whether MsLAC1 is capable of lignin polymerization *in planta*, several independent homozygous lines expressing *MsLAC1* under the control of the *AtLAC17* promoter (*pAtLAC17::MsLAC1*) or a corresponding control construct (dummy) in the *lac4–2 lac17* double mutant background were generated, and expression of *MsLAC1* was confirmed by qPCR (Fig. 7a). The semi-dwarf phenotype of the *lac4–2 lac17* mutants under constant light conditions [15] was fully reversed in two independent lines expressing *pAtLAC17::MsLAC1* (Fig. 7b). Furthermore, using HCl-phloroglucinol staining of stem cross sections, complete reversal of the irregular xylem phenotype of the inflorescence stems of the *lac4–2 lac17* double mutant was apparent in the lines complemented with *pAtLAC17::MsLAC1* (Fig. 7c).

**Fig. 8:**
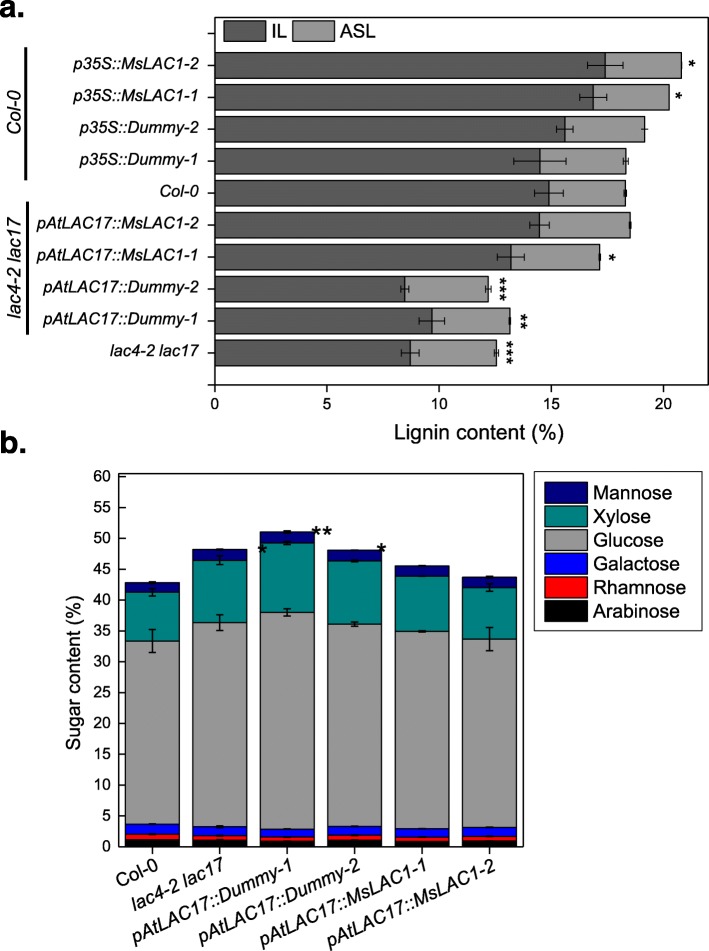
Lignin (**a**) and structural sugar contents (**b**) of mature stems from different *Arabidopsis* lines. Insoluble lignin (IL) and acid-soluble lignin (ASL) contents (% weight) were determined in mature stems of wild type, double mutant and complemented double, respectively. Plant materials from 3 to 4 plants were pooled as one replicate. Three replicates were then measured independently. Bars indicate mean ± SD of the replicates. Student’s t-test was used to determine significant differences for total lignin and sugar contents: *, P < 0.05; **, P < 0.01; ***, P < 0.001

To analyze lignin content and composition, dry stems of the *lac4–2 lac17* double mutant, *MsLAC1*-complemented plants, and the corresponding controls were subjected to lignin quantification and compositional analyses (Fig. 8a, Tables 1 and 2). As previously reported, the *lac4–2 lac17* double mutant has lower lignin content when compared to wild-type plants [15]. Under our growth conditions, total Klason lignin (KL) content of wild-type *Col-0* plants was 18.3% (3.4% acid soluble lignin [ASL] and 14.9% insoluble lignin [IL]). IL decreased to approximately 9% in the *lac4–2 lac17* double mutant, while ASL remained unchanged. In comparison, the negative control expressing the *pAtLAC17::Dummy* construct, displayed ASL and IL levels similar to the *lac4–2 lac17* double mutant. In the two independent *pAtLAC17::MsLAC1* lines, total lignin content was restored to 17.2 and 18.5%, respectively (Fig. 8a).

**Table 1:** Relative H, G, and S monomer contents in lignin from different *Arabidopsis* lines as determined by thioacidolysis

*Arabidopsis* lines	H%	G%	S%	S/G
*lac4–2 lac17*	2.34 ± 0.51	54.41 ± 1.39^a^	43.24 ± 1.89 ^a^	0.80 ± 0.06 ^a^
*pAtLAC17::Dummy-1*	2.15 ± 0.10	54.55 ± 1.21 ^a^	43.30 ± 1.24 ^a^	0.79 ± 0.04 ^a^
*pAtLAC17::Dummy-2*	2.44 ± 0.30	54.95 ± 1.38 ^a^	42.62 ± 1.46 ^a^	0.78 ± 0.05 ^a^
*pAtLAC17::MsLAC1–1*	1.21 ± 0.05	60.92 ± 2.04 ^c^	37.87 ± 2.08 ^b^	0.62 ± 0.06 ^b^
*pAtLAC17::MsLAC1–2*	0.94 ± 0.38	63.39 ± 0.57 ^bc^	35.67 ± 0.29 ^b^	0.56 ± 0.01 ^b^
*Col-0*	1.28 ± 0.18	64.26 ± 1.01 ^b^	34.46 ± 1.16 ^b^	0.54 ± 0.03 ^b^
*p35S::Dummy-1*	1.45 ± 0.45	64.28 ± 0.68 ^bc^	34.27 ± 1.13 ^b^	0.53 ± 0.02 ^b^
*p35S::Dummy-2*	1.81 ± 0.26	64.36 ± 0.22 ^bc^	33.83 ± 0.47 ^b^	0.53 ± 0.01 ^b^
*p35S::MsLAC1–1*	1.19 ± 0.72	70.63 ± 0.84 ^d^	28.18 ± 0.17 ^c^	0.40 ± 0.01 ^c^
*p35S::MsLAC1–2*	1.77 ± 0.29	68.52 ± 0.59 ^d^	29.71 ± 0.32 ^c^	0.43 ± 0.01 ^c^

Percentages for H, G and S monomers, and S/G ratios. All measurements were repeated three times. One-way ANOVA (followed by Tukey test) was used to determine significant differences for G%, S% and S/G ratios at *P* < 0.05. Samples with significant statistic difference are labelled with different letters

**Table 2:** Absolute contents of H, G, and S monomers in lignin of different *Arabidopsis* lines

*Arabidopsis* Line	H lignin	G lignin	S lignin	Total	Relative yield/%
*lac4–2 lac17*	2.3 ± 0.9	60.3 ± 5.6 ^a^	47.8 ± 1.1 ^a^	110.4 ± 7.6 ^a^	68.2 ± 0.2 ^a^
*pAtLAC17::Dummy*	1.7 ± 0.2	59.3 ± 0.4 ^a^	46.0 ± 2.7 ^a^	107.0 ± 2.6 ^a^	66.1 ± 0.1 ^a^
*pAtLAC17::MsLAC1*	2.3 ± 0.2	113.5 ± 6.2 ^b^	70.6 ± 6.4 ^b^	186.3 ± 10.4 ^b^	93.4 ± 0.2 ^c^
*Col-0*	1.8 ± 0.6	149.2 ± 30.2 ^b^	68.9 ± 8.0 ^b^	219.8 ± 38.6 ^b^	100 ^b^
*p35S::Dummy*	2.2 ± 0.2	150.8 ± 3.5 ^b^	58.9 ± 1.3 ^ab^	211.9 ± 4.8 ^b^	105.1 ± 0.1 ^b^
*p35S::MsLAC1*	3.2 ± 1.7	192.1 ± 16.3 ^c^	76.6 ± 5.2 ^b^	271.8 ± 20.2 ^c^	111.1 ± 0.2 ^d^

Yields of H, G and S monomers after thioacidolysis released from extract-free samples (i.e. after acetone extraction) are presented as μmol per gram sample. Relative yields are calculated based on total amount of monomer per gram of Klason lignin. Wild type (Col-0) was used as control and its yield arbitrarily set to 100%. All measurements were repeated three times. One-way ANOVA (followed by Tukey test) was used to determine significant differences at *P* < 0.05. Samples with significant statistic difference are labelled with different letters

The *Arabidopsis lac4–2 lac17* double mutant also displayed a change in the S/G monolignol ratio, increasing from 0.54 (wild type) to 0.80 (double mutant) (Table 1). More specifically, the monolignol composition of lignin in the stem tissue of the *lac4–2 lac17* mutant showed a 25% reduction in the S subunits, whereas the amount of G subunits decreased by 57% (Table 2). The negative controls (transformed with *pATLAC17::Dummy*) behaved like the double mutant, while complementation with *MsLAC1* (*pAtLAC17::MsLAC1* lines) partially restored wild type levels of S and G subunits (Tables 1 and 2). In association with the lignin modifications, the observed increase in structural carbohydrates in the *lac4–2 lac17* double mutant was also reversed by complementation with *MsLAC1* (Fig. 8b). Together, these observations clearly show that by expressing *MsLAC1* under control of the *AtLAC17* promoter in the *lac4–2 lac17* mutant, not only are growth-related phenotypes restored, but also the chemical and morphological attributes of the cell walls are largely restored.

### In *Arabidopsis*, ectopic expression of *MsLAC1* promotes interfascicular fiber development resulting in higher lignin content, lowered S/G ratio and shorter stems

Previous work had shown that over-expression of either *AtLAC4* or *AtLAC17* caused ectopic lignification, even in primary cell walls when exogenous monolignols are externally supplied [37]. To evaluate the effect of ectopic expression of *MsLAC1* on growth, *Arabidopsis* plants expressing *MsLAC1* under the control of the CaMV*35S* promoter were generated in the *Col-0* background. *MsLAC1* transcript levels were determined by qPCR, and two independent lines displaying high expression levels were selected for comparative analyses (Additional file 1: Figure S4). When plants ectopically expressing *MsLAC1* (*p35S::MsLAC1–1* and *p35S::MsLAC1–2*) were grown under constant light, both lignin content and composition in stem were affected. As shown in Fig. 8a, ASL content remained largely unchanged, but IL content increased from 14.9% in *Col-0* to 16.9 and 17.4% in *35S::MsLAC1–1* and *p35S::MsLAC1–2*, respectively (the two negative control lines were unchanged). Notably, the amount of G subunits was also increased in the *p35S::MsLAC1* lines, reducing the S/G ratio (Table 2). Conversely, sugar contents (i.e., glucose, xylose, mannose, galactose, rhamnose and arabinose) were not affected by ectopic expression of *MsLAC1* (Additional file 1: Figure S5).

**Fig. 9:**
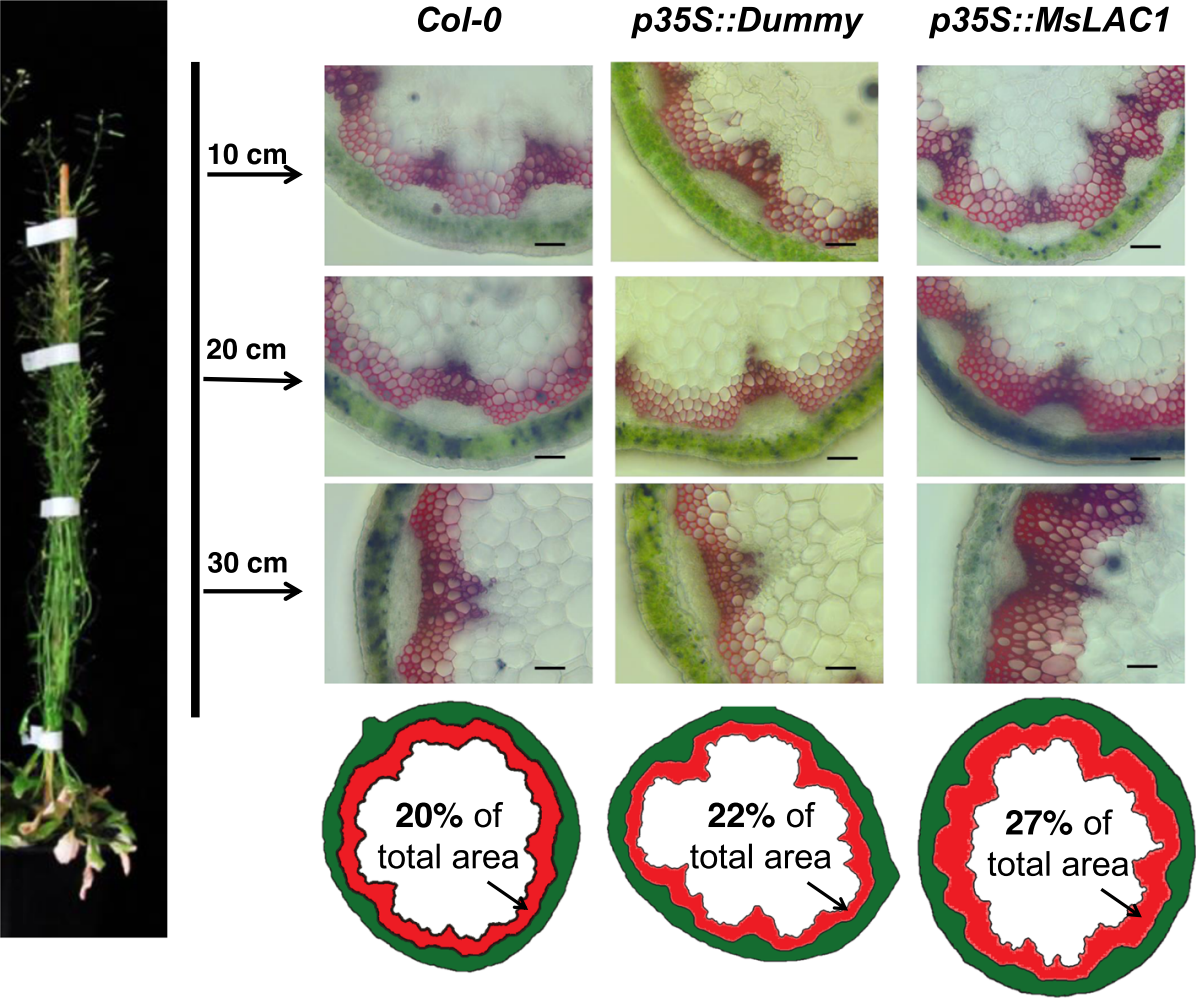
Increased stem lignification in *Arabidopsis* Col-0 ectopically expressing MsLAC1 under control of the 35S-promoter (*p35S::MsLAC1*). Plants were cultivated under continuous light (135 μmol/m^2^s). Stem cross sections were taken at 10 cm, 20 cm and 30 cm from tip and were stained with phloroglucinol-HCl. Scale bars: 100 μm. The imageJ-based diagrammatic representation indicated the area percentage of lignified xylem tissue of the entire cross-section (30 cm from tip)

**Fig. 10:**
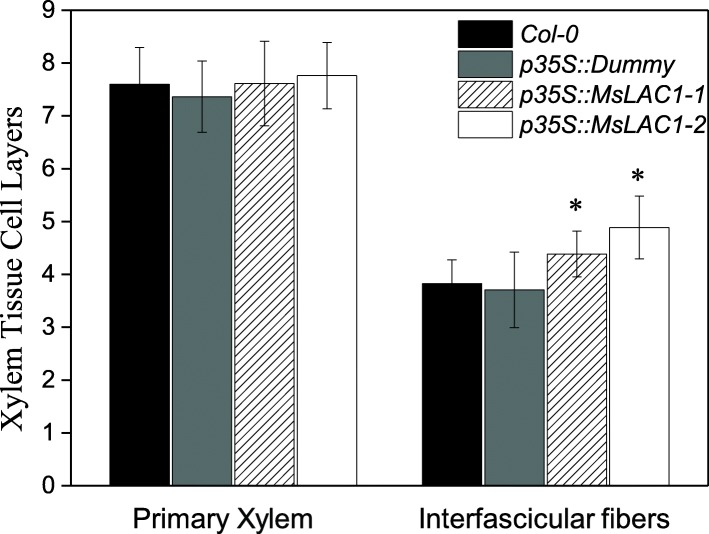
Quantification of primary xylem and interfascicular lignified cell layers in transgenic Arabidopsis plants. The analysis was based on cross-section of stem (30 cm from tip). Values are means ± SD for *n* = 15–20. Asterisks indicate significant differences (P < 0.05) relative to the wildtype determined with a single factor ANOVA

Plant growth of *p35S::MsLAC1–1* and *p35S::MsLAC1–2* lines under constant light was negatively affected when compared with *Col-0* wild type and empty vector control lines (Additional file 1: Figure S4). Previous studies have indicated that increased lignification (induced by over-expressing lignin-related TFs or enzymes involved in lignification) can impair plant growth [30, 38]. Thus, we hypothesized that the induced change in lignin content and composition in the *p35S::MsLAC1* lines were the cause for the observed shorter inflorescence stems (Additional file 1: Figure S4). Indeed, in mature *p35S::MsLAC1* plants (30 cm from tip), histochemical inspection indicated a 17.4–35.0% larger lignified xylem area than those for the corresponding controls (Fig. 9), and this is accompanied by an enhancement of interfascicular fiber formation. Specifically, 3–4 layers of interfascicular sclerified cells were observed in the cross-section area of WT and control lines, while plants overexpressing MsLAC1 displayed 4–6 layers (Fig. 10). These results corroborate the predicted function of MsLAC1 in lignification and development of interfascicular fibers, which, in turn, represses stem growth (Additional file 1: Figure S4).

## Discussion

Plant laccases [15, 19, 20, 22, 39] and peroxidases [14, 31] have been identified as key enzymes in lignin biosynthesis, playing a unique role in monolignol polymerization. For several plant species, both monocots and dicots, specific laccase isoforms have been shown to contribute to monolignol polymerization, affecting lignin content and lignin composition. The present study has extended this knowledge to *Miscanthus* as an important bioeconomy crop*,* providing not only a comprehensive functional analysis of a specific laccase isoform, MsLAC1, but also unraveling its specific effect on changing lignin composition, i.e., selectively promoting G unit deposition into lignin in both relative (decreasing S/G ratio) and absolute terms (G content). In agreement with previous work by Golfier et al. [9] and Hu et al. [8], our data further support the notion that the molecular network regulating secondary cell wall formation and lignification is largely conserved between dicots and monocots [40].

### Characterization of MsLAC1, a *Miscanthus* laccase isoform

The *Miscanthus* laccase isoform MsLAc1 has been identified as a potential key enzyme for monolignol polymerization based on i) its close sequence relationship with other monocot and dicot laccases known to be involved in lignin biosynthesis (Fig. 1, Additional file 1: Figure S1), ii) its co-expression with several genes of lignin biosynthesis, including genes of cognate transcription factors, in tissues (young stem internodes, leaf growth zones) known to undergo lignification (Figs. 2 and 3), iii) activation of the promoter region of its gene by transcription factors regulating secondary cell wall and lignin biosynthesis (Fig. 4, [9]; the strong induction of the *MsLAC1* promoter by MsSCM4, a transcription factor homologous to AtMYB58 and AtMYB63, both known to induce the expression of *AtLAC4* in *Arabidopsis* [30], iv) its targeting to the cell wall compartment (Fig. 5, with mCherry signals detected in cell wall after plasmolysis), and v) its in vitro ability to oxidize monolignols with a preference for sinapyl alcohol (Fig. 7, Additional file 1: Figure S3). In summary, these results suggested a role for MsLAC1 in the lignification process of *Miscanthus*.

### In *Arabidopsis*, MsLAC1 promotes lignification of interfascicular fibers, selectively promoting deposition of G subunits

In absence of available *Miscanthus* laccase mutants, the in vivo function of MsLAC1 was explored in *Arabidopsis* via complementation of the *Arabidopsis lac4–2 lac17* double mutant, which displays a clearly defined irregular xylem phenotype [15]. The rescue of the growth and collapsed xylem vessels phenotypes, together with the restored lignin content and composition in the complemented mutant (Figs. 7 and 8) confirm that MsLAC1 can functionally replace the combined activities of the *Arabidopsis* laccases AtLAC4 and AtLAC17. Beyond this observation, the conspicuous promotion of interfascicular fiber lignification after ectopically expressing MsLAC1 under control of the 35S-promoter (Figs. 8, 9 and 10, Additional file 1: Figure S4) may suggest that more G subunits were incorporated (Tables 1 and 2) into lignin of interfascicular fibers, enlarging the lignified xylem area (Fig. 9) and spatially extending the number of lignified cell layers (Fig. 10). Likewise, AtLAC17 was previously shown to be involved in the deposition of G subunits into lignin of interfascicular fibers [15].

In contrast to MsLAC1, a laccase from sugarcane (SofLAC) could only restore lignin content in an *Arabidopsis lac17* mutant, but not lignin composition [16]. Since the S/G ratio in the *lac17* mutant is restored to normal level in wild type after reintroduction of *AtLAC17,* it has been hypothesized that this laccase preferentially uses G units during polymerization [16]. However, MsLAC1, which displays a strong preference for S units (Fig. 7, Additional file 1: Figure S3), could also reduce the S/G ratio, i.e., from 0.78–0.82 in *lac4–2 lac17* mutant to 0.57–0.62 in complementation lines, and even to 0.4 in lines ectopically expressing MsLAC1. These results indicate that substrate availability at the site of lignification may be more important than enzyme affinity for different monolignol substrates. In addition, cross talk with peroxidases may be equally important to monolignol polymerization and may impact on final lignin composition. Interestingly, in *Arabidopsis*, the lignin polymerizing peroxidase AtPRX64 localized to the middle lamella, whereas AtLAC4 localized throughout the secondary cell wall layers [31], indicative of spatial control mechanisms.

Our results demonstrate that ectopic expression of MsLAC1 in *Arabidopsis* increases lignin content by 13.4–16.8%, suggesting that either sufficient endogenous monolignols were available or their biosynthesis was up-regulated via feedback loop. Likewise, in poplar stem ectopically expressing a cotton laccase, lignin content was increased by as much as 20% [41]. Note that since lignin thioacidolysis is based on cleavage of β-O-4 bonds, which are predominant in the secondary cell wall [42], the increased relative yield observed for the *p35S::MsLAC1* lines (Table 2) implies that more coniferyl and sinapyl residues were incorporated into lignin.

### The increase in G lignin does not result from the enzyme’s substrate preference but may be related to substrate availability

Unexpectedly, the in vitro enzyme activity of recombinant MsLAC1 protein (Fig. 7) was highest with sinapyl alcohol, while the complementation and ectopic expression experiments indicated an increased incorporation of G subunit into lignin, both in relative and absolute terms (Tables 1 and 2). This discrepancy suggests that the impact of MsLAC1 on monolignol polymerization was not simply the result of a preferred affinity towards sinapyl alcohol.

In *Arabidopsis*, the disruption of LAC4 and/or LAC17 downregulated several monolignol biosynthesis genes [15]. Based on feedback regulation models of biosynthesis and enzyme activity via metabolites, which can also affect the expression of the corresponding genes [43], accelerated consumption of monomers after over-expression of MsLAC1 may lead to increased biosynthesis of monolignol substrates. Alternatively, cell wall surveillance pathways might affect the expression of cell wall biosynthetic genes.

Considering that *Miscanthus* has a similar lignin biosynthesis pathway compared with *Arabidopsis* [8], MsLAC1 may also be involved in the lignification in *Miscanthus*. Therefore, changing the expression of MsLAC1 in *Miscanthus* via genetic manipulation may be an attractive strategy to modify the lignin composition while limiting the impact on the total lignin content, which in the end could be used for various end-uses in biorefinery applications [44]. In summary, while the stimulatory effects of MsLAC1 expression on the preferential incorporation of G subunits and the overall increase in total lignin content were highly significant, the exact molecular mechanisms leading to both phenomena remain to be elucidated.

## Conclusions

The expression of *MsLAC1*, a *Miscanthus* laccase isoform gene, is regulated by secondary cell wall MYBs and is involved in lignification of xylem fibers. MsLAC1 complements the lignification-deficient *Arabidopsis* double mutant *lac4–2 lac17*. Ectopic expression of MsLAC1 in *Arabidopsis* promotes interfascicular fiber development, resulting in higher lignin content and substantially increased amount of G-lignin, despite a preference for sinapyl alcohol of recombinant MsLAC1 protein in vitro.

## Methods

### Analysis of putative laccase isoforms in a *Miscanthus* transcriptome

Using more than 70 laccase protein sequences from both monocot and dicot plants, we performed a local tBLASTn search using the published *Miscanthus* transcriptome (Barling et al., 2013). More specifically, 17 laccase sequences from *Arabidopsis thaliana* (The Arabidopsis Information Resource [TAIR]; http://www.arabidopsis.org), 25 laccase sequences from *Sorghum bicolor* (Phytozome; http://www.phytozome.net/sorghum) and 29 laccase sequences from *Brachypodium distachyon* (Plant Genome and Systems Biology; http://pgsb.helmholtz-muenchen.de/plant/brachypodium) were used as queries.

Contigs containing full-length protein-coding sequences were further analyzed. Alignment of laccase sequences was performed using ClustalW alignment in MegAlign (DNASTAR, Madison, WI). A phylogenetic tree was generated using the Neighbour-Joining method (http://www.phylogeny.fr/) with bootstrap tests for 1000 replicates. Based on phylogeny and co-expression analyses, *MsLAC1* was selected as a putative *AtLAC17* ortholog.

To identify the promoter sequences of identified laccases, genomic DNA from *Miscanthus sinensis* (identification number: Sin-13) was sequenced to generate a partial genome database. The generated contig library was used to search for promoter sequences via BLAST search using the 5′-terminal sequences of laccases as query, and promoter sequences of corresponding genes were retrieved from the database.

### Plant material and growth conditions

*Miscanthus sinensis* seeds (identification number: Sin-13) were a gift of Iris Lewandowski and are descendant of plants originally collected in Honshu, Japan [45]. Plants were grown in the greenhouse as previously described [9]. For over-expression of *MsLAC1* in *Arabidopsis* Columbia-0 (*Col-0*) plants and complemented lines of the *Arabidopsis lac4–2 lac17* double mutant (gift from Dr. Richard Sibout, Institut Jean-Pierre Bourgin), were grown in soil under short-day conditions (21 °C, 8 h light/16 h dark, 110 μmolm^2^s) until rosette stage, and then transferred to long-day conditions (21 °C, 16 h light/8 h dark, 110 μmol/m^2^s) in the greenhouse. For phenotyping of transgenic *Arabidopsis* lines, plants were cultivated under continuous light in a growth chamber (21 °C, 60% relative humidity, 135 μmol/m^2^s).

### Histochemical staining of lignin

*Arabidopsis* inflorescence stems samples were embedded in 6% agarose and cut by hand using a razor blade [46]. Lignin was stained with HCl-phloroglucinol staining solution (2% phloroglucinol in absolute ethanol, mixed with equal volume of HCl before use). O-4-Linked coniferyl and sinapyl aldehydes in lignified cell walls were stained red [47] and images were captured using a Leica DM IRB inverted microscope.

### Tissue sampling, RNA extraction and quantitative RT-PCR

For 10-day to 3-month-old *Miscanthus* plants, different tissues (leaf, stem, and root) were separated and collected as a pool. 6-month-old *Miscanthus* stems and developing leaves were cut and dissected at their nodes, and seven internodes and five leaves were sampled. All samples were immediately frozen in liquid nitrogen.

After grinding, 30 mg or 50 mg of tissue from *Miscanthus* or *Arabidopsis*, respectively, were used for RNA extraction as previously described [9]. Subsequently, 0.5 μg of total RNA was reverse-transcribed by AMV reverse transcriptase (Roboklon) [48]. Transcript abundance was determined by quantitative RT-PCR using reference genes as described in [9]. Gene-specific primers used for *Miscanthus* are listed in Additional file 1: Table S2.

### Cloning of transcription factors and *MsLac1* promoter

Following the Gateway® cloning protocol (Fisher Scientific), the entry vector pDONR201 was used for initial cloning, and two different destination vectors, pART7 and pLuc, were used as effector and reporter plasmids, respectively, as previously reported [48].

Transcription factors *MsSND1* and *MsSCM2*–*4* were previously cloned [9], and used in this study. The promoter of *MsLAC1* as well as two additional transcription factors, *MsVND7* and *MsMYB52*, were cloned with gene-specific primers containing gateway overhangs (Additional file 1: Table S1). DNA fragments were cloned into pDONR201 via BP reaction according to the manufacturer’s instructions, subsequently sequenced and transferred into the corresponding destination vectors using the LR reaction.

### Promoter activation via dual luciferase assay

To determine activation of the *MsLAC1* promoter by transcription factors related to lignin biosynthesis, dual luciferase assays were employed using grapevine (*Vitis vinifera*) suspension cells, as previously described [48]. As indicated in Fig. 4, all transcription factor ORFs were expressed under control of the CaMV35S promoter in pART7, these plasmids being used as effectors. In the reporter plasmid, firefly luciferase was expressed under control of *MsLAC1* promoter (*pMsLAC1*); thus, the intensity of fluorescence indicated promoter induction via the respective transcription factors. Reporter plasmid, effector plasmid (and internal control, see below) were then coated onto gold particles and bombarded into grapevine suspension cells. After a two-day culture in the dark, bombarded cells were ground and fluorescence intensity of firefly and Renilla luciferase (internal control for transformation efficiency) in the isolated supernatants were quantified. The ratio between firefly and Renilla luciferase for each transfection experiment was normalized against the Renilla luciferase plasmid pRluc to represent the relative fold-activation of *pMsLAC1* via each transcription factors. All measurements were repeated three times (technical repeats), and all experiments were carried out independently at least twice.

### Sub-cellular localization of MsLAC1 protein by transient expression in *Nicotiana benthamiana* leaves

To determine the sub-cellular localization of the MsLAC1 protein, *Agrobacterium tumefaciens* ASE (pSOUP^+^) was transformed using the Greengate expression vectors [49] and subsequently infiltrated into leaves of 4-week-old *Nicotiana benthamiana* plants for transient expression of fluorescent protein-labelled MsLAC1 protein. Since an N-terminal signal peptide was predicted for the full-length MsLAC1 coding sequence, mCherry was fused to its C-terminus. Lt16b-GFP was used as a plasma membrane marker [33]. *Agrobacterium* strains containing this marker, mCherry tagged MsLAC1, and the p14 silencing inhibitor plasmid were dispersed in transformation buffer (10 mM MgCl_2_, 10 mM MES, 150 μM acetosyringone, pH 5.6) to an OD_600_ of 0.4, 0.4, and 0.1, respectively, and then mixed at equal volumes. After incubation at room temperature (RT) for 2 h, bacterial mixtures were co-infiltrated into tobacco leaves with a 2 ml sterile needle-less syringe. Dummy gene [49] replacing *MsLAC1* was used as a control construct that lacked the MSLAC1 coding sequence. Infiltrated leaves were collected after 3 days. To test whether MsLAC1 localizes to the cell wall, leaf discs were incubated in 30% sucrose solution for 2 h before microscopy to plasmolyze cell walls from the plasma membrane. Details regarding the Greengate constructs used in this study are presented in the legend of Fig. 5.

Images were captured using the Perkin-Elmer UltraView VoX spinning disk confocal mounted on a Leica DM16000 inverted microscope with a Hamamatsu 9100–02 CCD camera. The GFP filter (excitation 488 nm, emission 525 nm) was used to image GFP-tagged constructs. The RFP filter (excitation 561 nm, emission 595-625 nm) was used to image mCherry-tagged constructs. Samples were imaged using a Leica oil immersion 20× or 63× objective. All images were processed using Volocity image analysis software (Improvision).

### Heterologous expression of MsLAC1 protein in *Pichia pastoris*

The *Pichia pastoris* expression vector pPICZαA containing both MYC and HIS tags was used for heterologous expression of MsLac1 and CiFEH. The purified PCR products of *MsLAC1* and the vector pPICZαA were digested with *EcoRI* and *XbaI*, and then ligated at 4 °C overnight using T4 DNA ligase (Thermo Fisher Scientific, Catalog number: EL0014). Yeast extract Peptone Dextrose medium (YPD) containing 1% yeast extract, 2% peptone and 2% dextrose was used for general culture. Selected yeast strains were cultivated in buffered complex media containing glycerol (BMGY) and methanol (BMMY) for induction of protein expression. Competent yeast strain X33 cells were prepared fresh, and 2 μg of the ligated constructs was used for transformation. Positive colonies were selected on YPD plates containing 50 μg/mL Zeocin™, followed by colony PCR using the α-factor and the 3′ AOX1 Sequencing Primers (Additional file 1: Table S1). Colonies were then screened on minimal methanol histidine medium and minimal dextrose histidine medium plates supplemented with Zeocin™ for fast methanol utilization (Mut+) phenotype [34].

To check the expression and secretion of recombinant MsLAC1 protein, verified Mut + colonies were cultured in 5 mL BMGY medium in 50 mL tubes (30 °C, 180 rpm) and harvested by centrifugation (5000 rpm, 5 min), when cultures had reached an OD_600_ of 3.0. Pellets were washed and subsequently re-suspended in BMMY medium (supplemented with 0.3 mM CuSO_4_) and diluted to a final OD_600_ of 1.0 in 30 mL BMMY media in 300 mL flasks, cultured at the same conditions (30 °C, 180 rpm). Methanol (final concentration: 1%, v/v) was added every day to maintain inducing conditions. After the onset of induction, aliquots were collected daily by centrifugation (5000 rpm, 5 min). Supernatants were dialyzed against pH 5.0 20 mM sodium acetate buffer overnight at 4 °C (cold room) and stored for further analysis at − 80 °C. The pPICZαAfeh strain expressing recombinant fructan exohydrolase (FEH IIa) protein from *Cichorium intybus* was cultivated under the same conditions and used as a control.

### Measurement of laccase activity and protein concentration

Laccase activity was determined with ABTS (2,2-azino-bis3-ethylbenzothiazoline-6-sulfonic acid) as substrate [50]. Sample was diluted in pH = 5.0 50 mM HAc/NaAc buffer to 180 μL and mixed with 20 μL ABTS. 180 μL buffer without enzyme was mixed with ABTS in the same way and used as control. After incubation in 30 °C for 3 mins, absorbance at 420 nm was measured and the increased absorbance comparing with control (ΔA) was used for the calculation of enzyme activity:

$$ \mathrm{Laccase}\ \mathrm{activity}\ \left(\mathrm{U}\bullet {\mathrm{L}}^{-1}\right)=\frac{V_t\times N\times \Delta \mathrm{A}\times {10}^6}{V_e\times \varepsilon \times \Delta \mathrm{T}} $$

Where, V_t_, total volume; N, dilution fold; V_e_, sample volume; ε, Molar extinction coefficient for ABTS (3.6 × 104 M-1 cm-1); ΔT, time of incubation. To select the appropriate wavelength for each substrate, we measured substrate spectra at different concentrations. After calculating the correlation coefficients, we selected the wavelength with the highest absorbance for each substrate: 288 nm for synalpyl alcohol, P-coumaroyl alcohol, and coniferyl alcohol, as well as 296 nm for cinnamyl alcohol. One unit of enzyme activity was defined as the amount of enzyme that oxidizes 1 μmol substrate per minute at 30 °C. Protein concentration was determined via the Bradford assay using a calibration curve based on bovine serum albumin (0.1–1 mg/mL) with Bradford Reagent from Sigma (Product number: B6916).

### Enzymatic oxidation of monolignols by recombinant MsLAC1 protein

Cinnamyl alcohol (CAS No.: 104–54-1), *p*-coumaryl alcohol (CAS No.: 3690-05-9), coniferyl alcohol (CAS No.: 458–35-5), and sinapyl alcohol (CAS No.: 537–33-7) were used as substrates to evaluate the ability of recombinant MsLAC1 protein to oxidize the canonical monolignols. In the assay, 0.02 U enzyme was mixed with 0.5 mM of each substrates [51]. Reactions were performed in 1.5 mL reaction vials in 50 mM NaAc-HAc buffer, pH 5.0, at 30 °C. Absorbance of the reaction mixtures were monitored between 250 to 650 nm [52]. For sinapyl alcohol, the reaction mixture was scanned at 5, 10, 20, 30 and 60 min after reaction start, respectively, whereas for other monolignols reaction mixtures were scanned at 0.5, 1, 2, 6, 12 h, respectively. The reaction was terminated by adding two drops of 10 mM NaN_3_. Water replacing the recombinant MsLAC1 protein was used as the control in different mixtures and measured in the same time periods.

### Native-PAGE, SDS-PAGE and Western blot analysis

Recombinant protein samples were mixed with Roti®-Load 1 or Roti®-Load 2 followed by incubation at 100 °C for 5 min, for SDS-PAGE and Native-PAGE, respectively. Both types of electrophoresis were performed on a 4.5% stacking gel and a 12% separating gel, using chambers from Bio-Rad®. Sample loading, running conditions and ABTS staining procedures were as previously described [51].

After SDS-PAGE, proteins were electroblotted onto an Immobilon-P PVDF membrane (Sigma-Aldrich). Recombinant MsLAC1 protein was detected by Western Blot analysis, using anti-MYC (Thermo Fisher Scientific, MA1–980) as a primary antibody, detected by using SuperSignal West Dura Extended Duration Substrate for HRP (Thermo Fisher Scientific, Catalog number: 34075) after incubation with anti-mouse IgG secondary antibody (Bio-Rad, Catalog number: 172–1011).

### Generation of transgenic *Arabidopsis* lines

All transgenic plant lines were generated using the *Agrobacterium tumefaciens* strain ASE (pSOUP^+^) transformed with Greengate constructs using the floral dip method [53]. The *Arabidopsis lac4–2 lac17* double mutant was complemented with the *pAtLAC17::MsLAC1* construct (i.e.*,* expressing *MsLAC1* under control of the *AtLAC17* promoter). In addition, MsLAC1 was ectopically expressed in *Arabidopsis Col-0* plants, using a *p35S::MsLAC1* construct. The Greengate constructs used for over-expression are depicted in Additional file 1: Figure S4. For analysis of complementation and ectopic expression homozygous F3 plants were used.

### Lignin analysis

Mature stems of *Arabidopsis* were dried and all siliques and leaves removed. Approximately 12 cm long stem segments were ground and then extracted with hot acetone in a Soxhlet column overnight to remove soluble compounds. The contents of acid soluble lignin (ASL) and insoluble lignin (IL) were measured according to [54]. Neutral cell wall carbohydrate contents were determined via liquid chromatography [55], while lignin composition was determined by gas chromatography after thioacidolysis as previously described [56]. All measurements were done in technical triplicates, using two independent transgenic lines.

### Accession numbers

All sequence data from this paper can be found in GenBank under the following accession numbers. Promoter sequences: *pMsLAC1* (MK310212). Coding sequences: *MsLAC1* (MK310209), *MsSND1* (KY930620), *MsVND7* (MK310211), *MsSCM2* (MF996502), *MsSCM3* (KY930622), *MsSCM4* (MF996501), *MsMYB52* (MK310210).

### Statistical treatment of data

Three independent experiments were carried out for all measurements. The SD value indicates the standard deviation calculated from the mean. For statistical analysis, the Student’s T-test was performed, and asterisks were used to represent the significant differences (*, *P* < 0.05; **, *P* < 0.01;***, *P* < 0.001). One-way ANOVA followed by Tukey test was also used to determine significant differences for lignin composition.

## Supplementary information

**Additional file 1:** **Table. S1.** Primers used for cloning and colony PCR in this study. **Table. S2.** Primers used for quantitative real-time PCR analysis. **Figure S1.** Phylogenetic tree including putative laccases of *Miscanthus* and laccase proteins from *Brachypodium distachyon*. **Figure S2.** Multiple sequence alignment of MsLAC1 with other laccase proteins known to be involved in lignification. **Figure S3.** Time course of spectral change in reaction mixtures containing cinnamyl, *p*-coumaryl or coniferyl alcohol, respectively, co-incubated with purified rMsLAC1 protein. **Figure S4.** Under long-day condition, ectopic expression of MsLAC1 (*p35S::MsLAC1*) in *Arabidopsis* ecotype *Col-0* reduces stem growth. **Figure S5.** Structural sugar contents in different *Arabidopsis* lines after ectopic expression of MsLAC1 (*p35S:MsLAC1*, see **Figure S4**).

## Data Availability

All data generated or analyzed during the study are included in this manuscript and Additional file.

## Abbreviations

ABTS: 2,2-azino-bis3-ethylbenzothiazoline-6-sulfonic acid
ASL: Acid Soluble Lignin
CCoAOMT: Caffeoyl-CoA O-methyltransferase
G: Guaiacyl
H: *p*-Hydroxyphenyl
HCT: Hydroxycinnamoyltransferase
IL: Insoluble Lignin
KL: Klason Lignin
LAC: Laccase
PRX: Peroxidase
S: Syringyl
SCM: Secondary Cell wall MYB protein
SMRE: Secondary wall MYB Responsive Element
SNBE: Secondary cell wall NAC Binding Element
SND1: Secondary wall-associated NAC Domain protein 1
VND7: Vascular-related NAC-Domain protein 7
