# Supplementary material for: The in vivo impact of MsLAC1, a Miscanthus laccase isoform, on lignification and lignin composition contrasts with its in vitro substrate preference
Source: BMC Plant Biol. 2019 Dec 12;19:552. doi: 10.1186/s12870-019-2174-3 (PMC6909574; doi:10.1186/s12870-019-2174-3)
Supplement: Supplementary file 1 — Additional file 1: Table. S1. Primers used for cloning and colony PCR in this study. Table. S2. Primers used for quantitative real-time PCR analysis. Figure S1. Phylogenetic tree including putative laccases of Miscanthus and laccase proteins from Brachypodium distachyon. Figure S2. Multiple sequence alignment of MsLAC1 with other laccase proteins known to be involved in lignification. Figure S3. Time course of spectral change in reaction mixtures containing cinnamyl, p-coumaryl or coniferyl alcohol, respectively, co-incubated with purified rMsLAC1 protein. Figure S4. Under long-day condition, ectopic expression of MsLAC1 (p35S::MsLAC1) in Arabidopsis ecotype Col-0 reduces stem growth. Figure S5. Structural sugar contents in different Arabidopsis lines after ectopic expression of MsLAC1 (p35S:MsLAC1, see Figure S4). [file 12870_2019_2174_MOESM1_ESM.docx]

**Additional Information**

**The *in vivo* impact of MsLAC1, a *Miscanthus* laccase isoform, on lignification and lignin composition contrasts with its *in vitro* substrate preference**

Feng He^1^, Katja Machemer-Noonan^1^, Phillipe Golfier^1^, Faride Unda^2^, Johanna Dechert^1^, Wan Zhang^1^, Natalie Hoffmann^3^, Lacey Samuels^3^, Shawn D. Mansfield^2^, Thomas Rausch^1*^ and Sebastian Wolf^1*^

^1^Centre for Organismal Studies (COS) Heidelberg, Heidelberg University, Heidelberg, Germany

^2^Department of Wood Science, University of British Columbia, Vancouver, Canada

^3^Department of Botany, University of British Columbia, Vancouver, Canada

*Corresponding authors:

Prof. Dr. Thomas Rausch Dr. Sebastian Wolf

COS Heidelberg COS Heidelberg

Im Neuenheimer Feld 360 Im Neuenheimer Feld 230

D-69120 Heidelberg D-69120 Heidelberg

Germany Germany

Fon: +49(0)6221-546621 Fon: +49 6221 54-5614

Fax: +49(0)6221-545659 Fax: +49(0)6221-545659

[thomas.rausch@cos.uni-heidelberg.de](mailto:thomas.rausch@cos.uni-heidelberg.de) [sebastian.wolf@cos.uni-heidelberg.de](mailto:sebastian.wolf@cos.uni-heidelberg.de)

**Table. S1 Primers used for cloning and colony PCR in this study**

| **Primer** | **Sequence** | **Aim** |
| --- | --- | --- |
| PicMsLAC1_F | ccgGAATTCATGGCGTCCTCGTCTGC | Cloning of MsLAC1 into pPICZαA. |
| PicMsLAC1_R | tgcTCTAGATAGCACTGAGGCAGATCCAAT |  |
| PicCiFEHIIa | ACTAATGAATTCCAGCAGATTGAACAGCCG | Cloning of CiFEHIIa into PICZαA |
| PicCiFEHIIa | TACTAATTCTAGATCTGCTGCACTTTTTACATTCTG |  |
| pPICZαA_F | TCTCTCGAGAAAAGAGAGGCTG | Colony PCR |
| PICHIA_REV2 | AGGAACAGTCATGTCTAAGGCTAC |  |
| GwpMsLAC1_F | GGGGACAAGTTTGTACAAAAAAGCAGGCTTCAGGATAAGGTAAGGGCAAATAT | Cloning of MsLAC1 promoter into Gateway vector for DLA assay. |
| GwpMsLAC1_R | GGGGACCACTTTGTACAAGAAAGCTGGGTTAATTGGTAGCACGGGGGTA |  |
| GwMsVND7_F | GGGGACAAGTTTGTACAAAAAAGCAGGCTCCATGGATCAGCAGGAGGAGTCT | Cloning of MsVND7 into Gateway vector for DLA assay. |
| GwMsVND7_R | GGGGACCACTTTGTACAAGAAAGCTGGGTTCACTGCAACAAGGATCCC |  |
| GwMsMYB52_F | GGGGACAAGTTTGTACAAAAAAGCAGGCTCCTCGAGGATGTGCACGAGG | Cloning of MsMYB52 into Gateway vector for DLA assay. |
| GwMsMYB52_R | GGGGACCACTTTGTACAAGAAAGCTGGGTTCACTGACGATGAGAAGCCC |  |
| GGpAtLAC17_F | AACAGGTCTCAacctGACTACACTTTACTACGTAGTAAT | Cloning of AtLAC17 promoter into Greengate module A. |
| GGpAtLAC17_R | AACAGGTCTCTtgttGTGTCCAAGCTCACTTCA |  |
| GGMsLAC1_F | AACAGGTCTCAggctCAATGTCAAGCTCAAGCAGCAG | Cloning of MsLAC1 into Greengate module C. |
| GGMsLAC1_R | AACAGGTCTCTctgaGCAGACCGGCAGGTCAG |  |

**Table. S2 Primers used for quantitative real-time PCR analysis**

|  | **Primer** | **Sequence** | **Length of product** | |
| --- | --- | --- | --- | --- |
| **Laccase genes** | qMsLac1_F | GCCGAGACGAGGAAGTACCAGT | | 184 |
|  | qMsLac1_R | CGTGCCAGTGGATGCTGATGTT | |  |
| **Transcription factors** | MsSND1_F | GACATCCAAGAGAAGTGCCG | | 84 |
|  | MsSND1_R | CGTCGGGTACTTCTTGTCCT | |  |
|  | MsSCM4_F | ACATAGCAAGCTTCAGCCCA | | 105 |
|  | MsSCM4_R | CCACCAGGAGTTCAGGTTCC | |  |
|  | MsSCM3_F | GCAGCCCTACGGAATCGA | | 93 |
|  | MsSCM3_R | CCAGCGGGTCTTGGTCAT | |  |
|  | MsSCM2_F | AAGGCAGCTTCCTCACAGTC | | 132 |
|  | MsSCM2_R | GCTGGACTGCTCCGATGAAT | |  |
| **Monolignol**  **biosynthesis** | MsHCT_F | GGAGCACTGGATAGGATGGA | | 161 |
|  | MsHCT_R | AAGTCGGCATCATGGATAGG | |  |
|  | MsCCoAOMT_F | ACGCCGACAAGGACAACTAC | | 155 |
|  | MsCCoAOMT_R | GTCACGGTAGAAGCGGATGT | |  |
| **Reference genes** | MsPP2A_F | GCTAGCTCCTGTCATGGGTC | | 89 |
|  | MsPP2A_R | TCATGTTCGGAACCCTGTCC | |  |
|  | MsUBC_F | CTGAACCAGACAGCCCACTT | | 63 |
|  | MsUBC_R | CTCTGATATCACCCGACCGC | |  |
|  | AtPDF2_F | TAACGTGGCCAAAATGATGC | | 61 |
|  | AtPDF2_R | GTTCTCCACAACCGCTTGGT | |  |

MsPP2A: *Protein phosphatase 2A subunit 3A* in *Miscanthus*; MsUBC: *peroxin 4* in *Miscanthus*. AtPDF2: Protodermal Factor 2 in *Arabidopsis.*

**Fig. S1**

**Phylogenetic tree including putative laccases of *Miscanthus* and laccase proteins from *Brachypodium distachyon***

Sequences were aligned with Mega 5 using the Neighbor-joining method. Subgroup designation was based on Wang et al., (2015). Note that primers for amplification of the MsLAC1 open reading frame were designed according to the Mxg_TContig47643 sequence (marked with dot), which clustered in subgroup 4 together with *BdLAC5* and *BdLAC6*, the latter two laccases known to be involved in lignification (Wang et al., 2015). The percentage of replicate trees in which the associated taxa clustered together in the bootstrap test (1000 replicates) is displayed next to the branches. The tree is drawn to scale, with branch lengths in the same units as those of the evolutionary distances used to infer the phylogenetic tree.


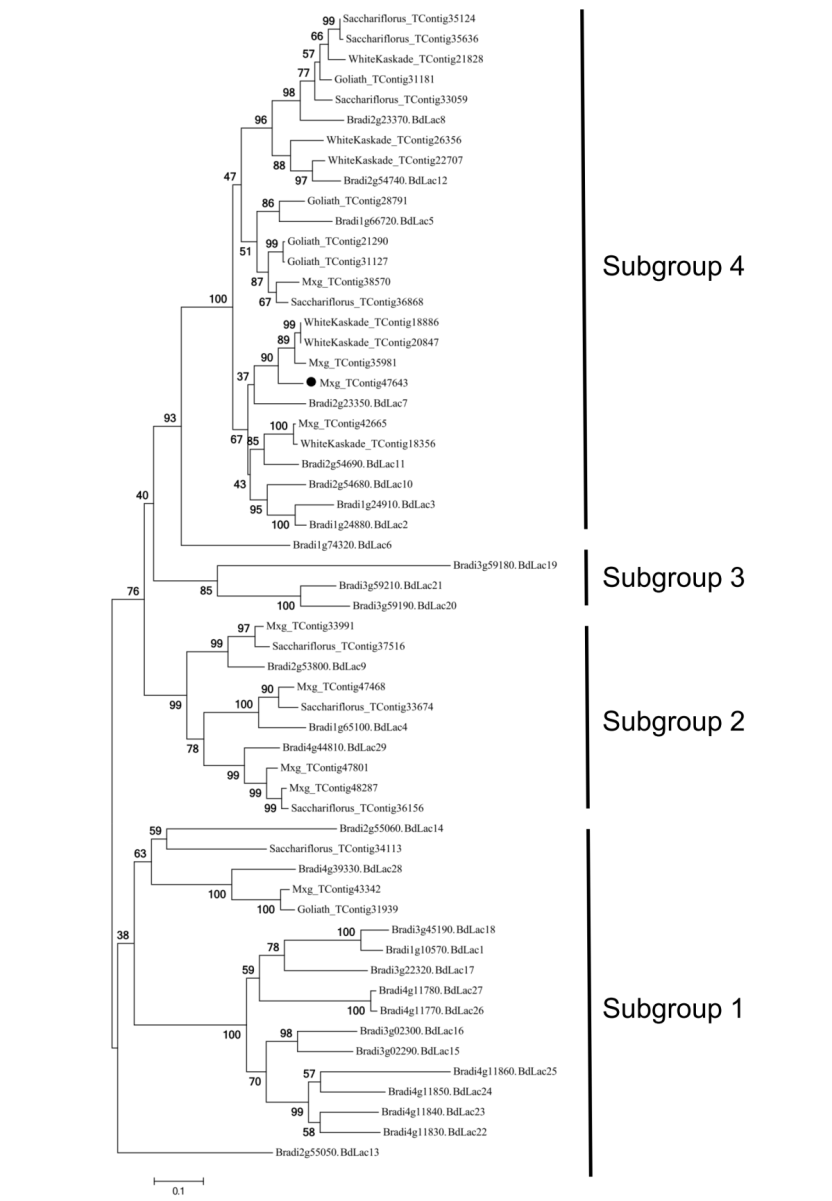


**Fig. S2**

**Multiple sequence alignment of MsLAC1 with other laccase proteins known to be involved in lignification**

Amino acid sequences of *Miscanthus* (MsLAC1), *Brachypodium* (BdLAC5), sugarcane (SofLAC), poplar (PtLAC3) and *Arabidopsis* (AtLAC4 and AtLAC17) laccases were analyzed with Mega 5 and then aligned with ClustalW software. Conserved copper-binding sites are underlined in red. The blue triangle marks the predicted cleavage site for the signal peptide of MsLAC1.


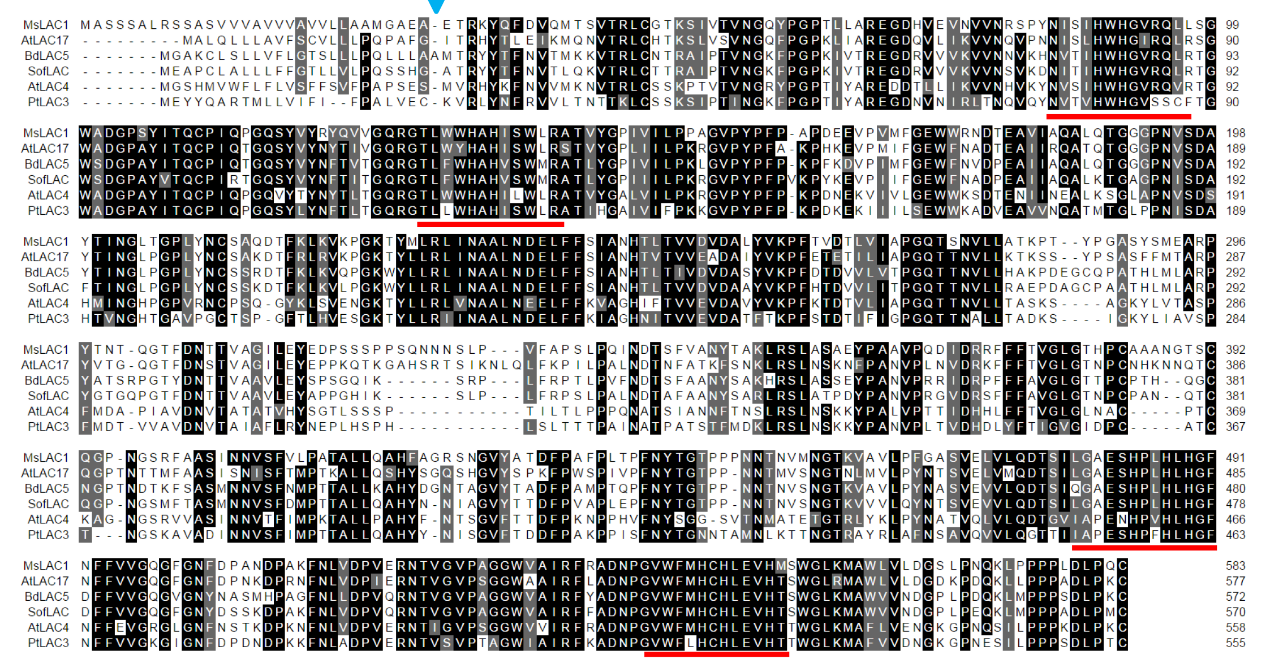


**Fig. S3**

**Time course of spectral change in reaction mixtures containing cinnamyl, *p*-coumaryl or coniferyl alcohol, respectively, co-incubated with purified rMsLAC1 protein**

Monolignols (final concentration 1 mM) were incubated with 0.002 U recombinant MsLAC1 protein (rMsLAC1) in 50 mM acetate buffer, pH 3.0. Spectra of reaction mixtures (250 nm – 650 nm) were recorded at indicated time intervals (control: with substrate only); time courses of absorption change at 280 nm are also displayed. Initial absorbance at 280 nm was set to 100%.


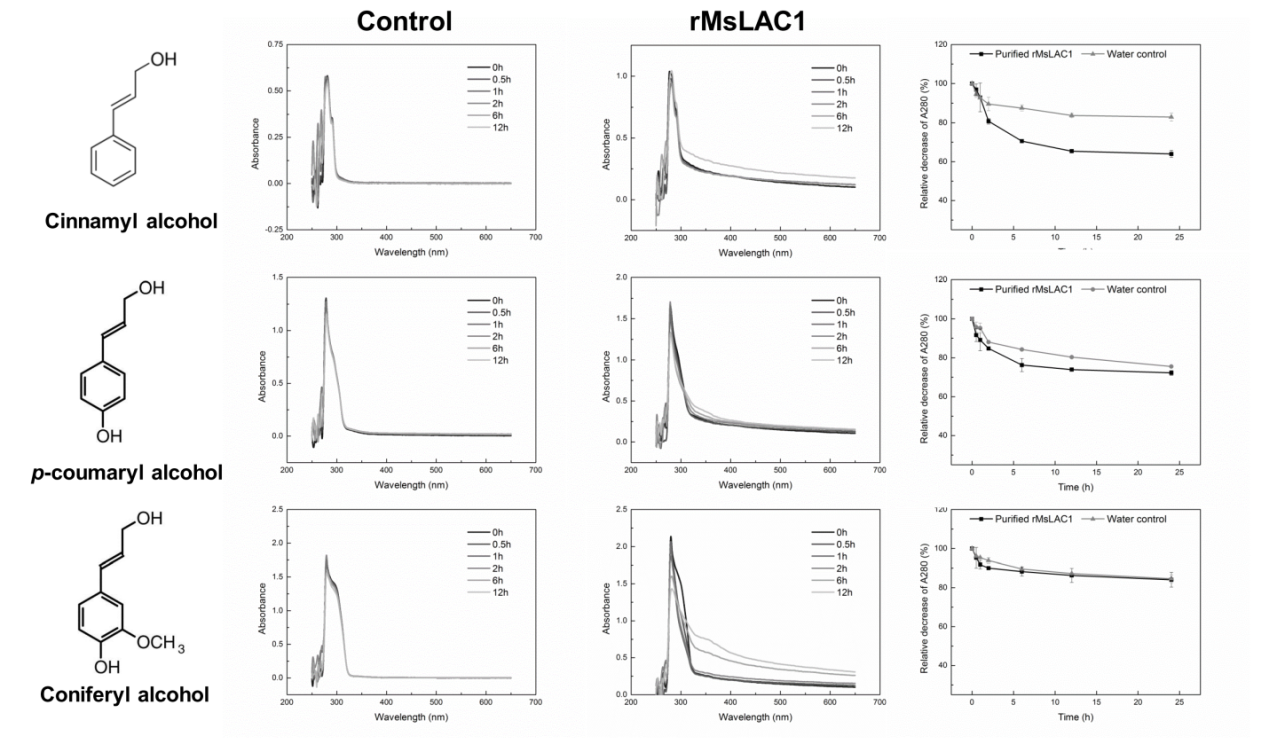


**Fig. S4**

**Under long-day condition, ectopic expression of MsLAC1 (*p35S::MsLAC1*) in *Arabidopsis* ecotype *Col-0* reduces stem growth**

(a) Schematic representation of *p35S::MsLAC1* Greengate construct and the corresponding *p35S::Dummy* control used for ectopic expression. (b) Confirmation of *MsLAC1* expression by qPCR analysis (*AtPDF2* was used as reference gene). (c) Phenotypes of 8-week-old plants.


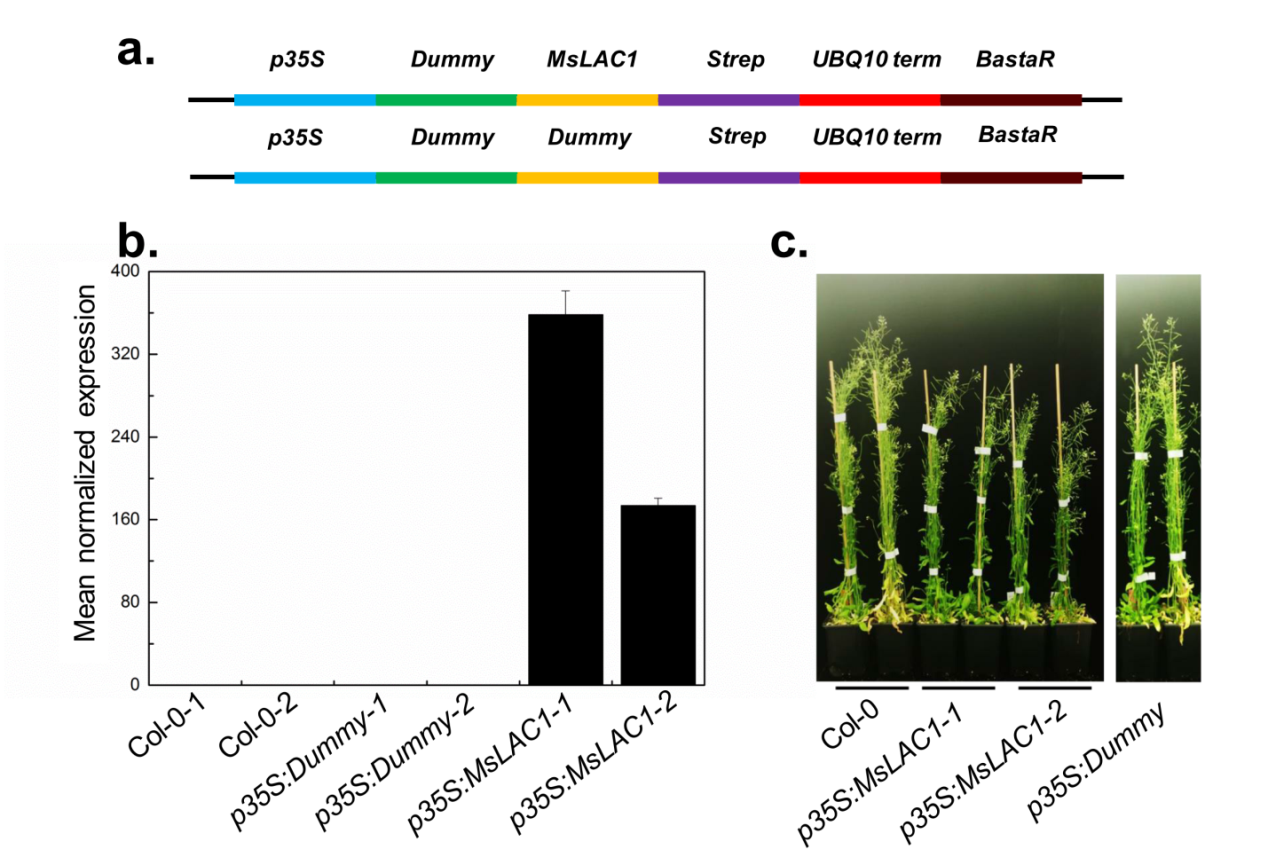


**Fig. S5**

**Structural sugar contents in different *Arabidopsis* lines after ectopic expression of MsLAC1 (*p35S:MsLAC1*, see Fig. S4)**

Bars indicate mean ± SD of three technical replicates. Student’s t-test revealed no significant differences.

**
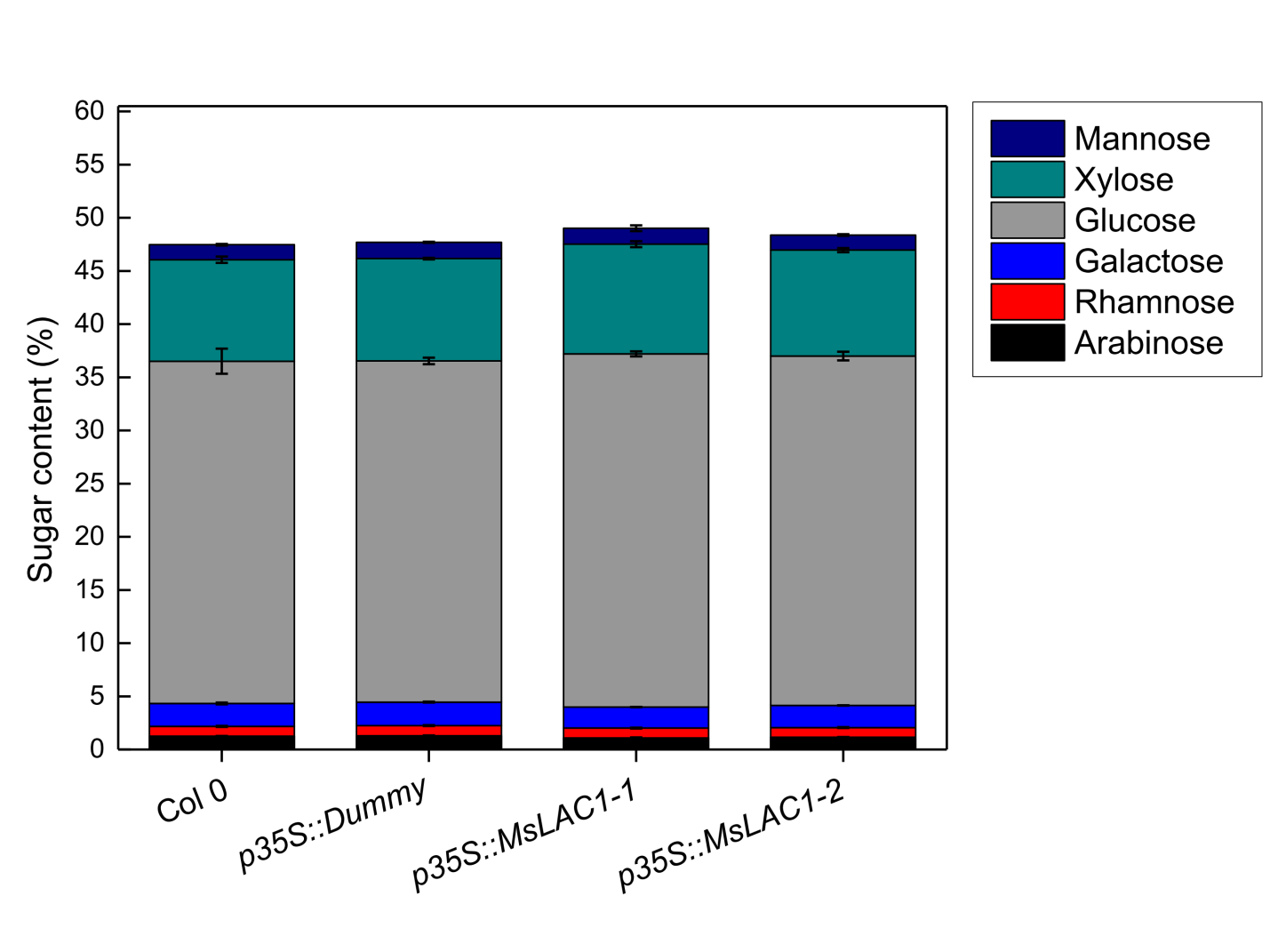
**
